# Therapeutic Anti-Fibrotic Effects of a Dual Hyaluronic Acid Hybrid Complex in Bleomycin-Induced Dermal Fibrosis and UVB-Irradiated Human Skin

**DOI:** 10.3390/ijms27073038

**Published:** 2026-03-26

**Authors:** Hyojin Roh, Ngoc Ha Nguyen, Jinyoung Jung, Jewan Kaiser Hwang, Young In Lee, Yujin Baek, Inhee Jung, Jihee Kim, Ju Hee Lee

**Affiliations:** 1 Department of Dermatology & Cutaneous Biology Research Institute, Yonsei University College of Medicine, Seoul 03722, Republic of Koreanguyenngocha7996@gmail.com (N.H.N.);; 2 Mymirae Dermatologic Clinic, Seoul 07326, Republic of Korea; 3 Department of Dermatology, University of Medicine and Pharmacy at Ho Chi Minh City, Ho Chi Minh City 700000, Vietnam; 4 Mymirae Research Institute for Dermatologic Science, Seoul 07326, Republic of Korea; 5 Scar Laser and Plastic Surgery Center, Yonsei Cancer Hospital, Seoul 03722, Republic of Korea; 6 Global Medical Research Center Co., Ltd., Seoul 06526, Republic of Korea; 7 Department of Dermatology, Yongin Severance Hospital, Yonsei University College of Medicine, Yongin-si 16995, Republic of Korea

**Keywords:** skin fibrosis, hyaluronic acid, bleomycin, oxidative stress, inflammation

## Abstract

Cutaneous fibrosis is characterized by aberrant wound healing with excessive extracellular matrix deposition, sustained inflammation, and oxidative stress, while currently available therapies show limited efficacy and safety. A Dual Hyaluronic Acid Compound (DHC), consisting of high-molecular-weight, low-molecular-weight, and minimally cross-linked hyaluronic acid, has demonstrated regenerative and antioxidant properties, but its anti-fibrotic effects have not been fully explored. This study investigated the anti-fibrotic potential of DHC using a bleomycin-induced murine dermal fibrosis model and a UVB-irradiated ex vivo human skin model. In C57BL/6 mice, dermal fibrosis was induced by daily bleomycin injections for three weeks, followed by intradermal DHC administration. Histological and biomechanical analyses showed that DHC significantly reduced dermal thickness, collagen deposition, and skin hardness compared with untreated fibrotic controls. DHC decreased α-SMA expression and increased MMP1 levels, indicating attenuation of myofibroblast activation and enhanced matrix remodeling. It also reduced macrophage markers (CD68, CD163) and pro-inflammatory cytokines (IL-1β, TNF-α). Furthermore, DHC restored superoxide dismutase (SOD) and catalase (CAT) activity and upregulated NRF2, HO-1, and NQO1 expression in the in vivo model. Similarly, DHC upregulated SOD and CAT activity and reduced pro-inflammatory cytokines (IL-6, TNF-α) in the ex vivo human skin model. These findings suggest that DHC exerts multimodal anti-fibrotic effects through coordinated regulation of fibroblast activation, inflammation, and oxidative stress, supporting its potential as a therapeutic approach for cutaneous fibrosis.

## 1. Introduction

Wound healing is a tightly regulated, multistage process consisting of hemostasis, inflammation, proliferation, and remodeling [1]. Hemostasis occurs within minutes to hours after injury, as platelet activation and fibrin clot formation stop bleeding and create a provisional matrix [1,2]. The inflammatory phase (hours to days) follows, with neutrophils clearing debris and pathogens and pro-inflammatory M1 macrophages releasing cytokines to coordinate repair [1,3,4]. During proliferation (days to weeks), fibroblasts deposit collagen-rich granulation tissue, angiogenesis is stimulated largely by Vascular Endothelial Growth Factor (VEGF), keratinocytes restore the epidermal barrier, and macrophages shift toward the pro-reparative M2 phenotype [1,3,5]. Finally, in remodeling (weeks to months), inflammation resolves and collagen III is replaced by stronger, organized collagen I, leading to scar maturation and improved tensile strength [1,3,4].

Skin fibrosis arises from dysregulated wound healing and chronic inflammation, leading to excessive extracellular matrix (ECM) deposition and dermal scarring [6]. In fibrotic skin conditions (e.g., hypertrophic scars, keloids, scleroderma), persistent inflammatory signals, such as interleukin (IL)-1 and Tumor Necrosis Factor-alpha (TNF-α), recruit immune cells (macrophages, neutrophils, lymphocytes) that secrete pro-fibrotic cytokines (e.g., Transforming Growth Factor-beta (TGF-β), Platelet-Derived Growth Factor (PDGF), IL-4/13) and reactive oxygen species (ROS). These factors drive sustained fibroblast-to-myofibroblast activation, resulting in overproduction of collagen and other ECM proteins [3]. Oxidative stress itself is a key amplifier of this cycle, as ROS promote collagen synthesis and perpetuate fibroblast activation in skin fibrosis [7,8]. The result is a vicious loop of chronic inflammation, immune-cell infiltration, ROS generation, and progressive dermal thickening due to collagen-rich ECM accumulation [9].

Current therapies for cutaneous fibrosis are largely empirical and palliative. Intralesional corticosteroid injections are commonly used to suppress inflammation and fibroblast proliferation [10], and systemic immunosuppressants (e.g., methotrexate, mycophenolate mofetil) are employed in diffuse scleroderma [11], but these often carry risks of skin atrophy [10] or systemic toxicity [12]. Physical modalities such as silicone sheeting and energy-based devices (pulsed-dye or fractional lasers) can remodel scar collagen and improve appearance, but outcomes are variable and typically require combined therapies and multiple sessions [13]. In general, no single established therapy completely reverses skin fibrosis, and many patients experience recurrence or incomplete response despite combined treatment algorithms. Thus, novel anti-fibrotic strategies with improved efficacy and safety are needed.

Our Dual Hyaluronic acid Compound (DHC) is an engineered formulation combining non-cross-linked high-molecular-weight HA (HMW-HA), non-cross-linked low-molecular-weight hyaluronic acid (LMW-HA), and a minimally cross-linked HA hybrid [14]. Each component contributes distinct bioactivities that may counteract fibrosis. HMW-HA is a high-viscosity, hydrophilic polymer that binds water and maintains tissue hydration, and it is known to dampen inflammatory signaling [15]. In contrast, LMW-HA fragments have potent antioxidant properties [16]. The minimally cross-linked HA hybrid provides structural stability and mimics a soft ECM scaffold, potentially enhancing mechanical support and modulating fibroblast mechanotransduction [17,18]. Together, these HA components are hypothesized to synergize by increasing dermal hydration, inhibiting inflammation, neutralizing oxidative stress, and remodeling the ECM in fibrotic tissue.

Despite these theoretical benefits, the anti-fibrotic effects of DHC in skin have not been tested. To address this knowledge gap, we evaluated DHC in preclinical fibrosis models. The in vivo study was conducted to evaluate the therapeutic effects of DHC on bleomycin-induced dermal fibrosis, inflammation, oxidative stress, and biomechanical skin properties under a physiologically relevant fibrotic microenvironment. The ex vivo human skin model was employed to isolate tissue-level responses to ultraviolet B (UVB)-induced inflammatory and oxidative damage and to validate antioxidant-related effects of DHC in human skin.

## 2. Results

### 2.1. In Vivo Study on Bleomycin-Induced Fibrotic Mouse Skin

#### 2.1.1. Reduction in Dermis Thickness, Collagen Fiber Production, and Skin Hardness

**Figure 1:**
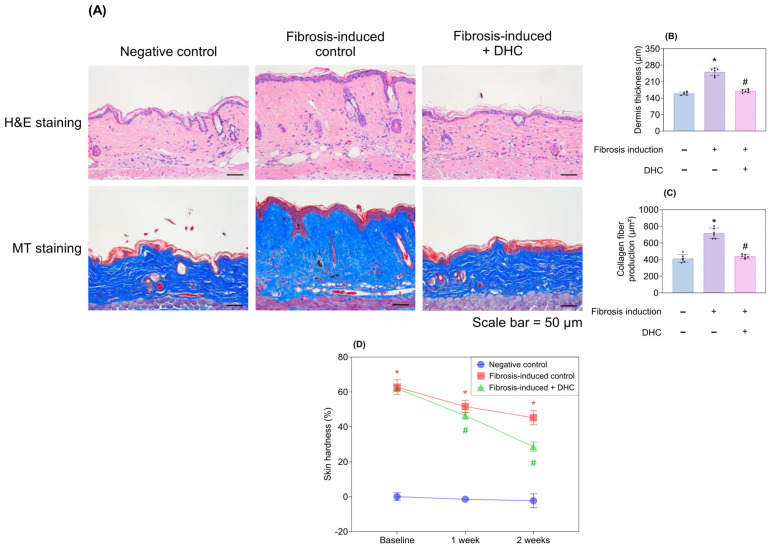
Reduction in dermis thickness, skin hardness, and collagen production by DHC in the in vivo mouse model. (**A**) H&E staining (nuclei: blue/purple; cytoplasm: pink) and MT staining (collagen: blue; cytoplasm: red) images of the negative control, fibrosis-induced control, and fibrosis-induced + DHC groups. Quantitative analysis of (**B**) dermal thickness and (**C**) collagen fiber production, corresponding to the statistical analysis of (**A**), measured after 3 weeks of fibrosis induction followed by 2 weeks of treatment with normal saline or DHC. (**D**) Skin hardness significantly decreased after DHC treatment at 1 and 2 weeks compared with normal saline treatment (*p* < 0.05). Data are presented as mean ± SD; dots represent individual values. * *p* < 0.05 vs. negative control; # *p* < 0.05 vs. fibrosis-induced control. H&E, Hematoxylin and Eosin; MT, Masson’s trichrome.

Fibrosis induction led to a visually significant increase in dermis thickness and collagen fiber quantity after 3 weeks via histological assessment, and DHC markedly mitigated these effects (Figure 1A). Quantitative analysis confirmed these findings, showing a statistically significant reduction in fibrosis-induced dermis thickness and collagen fiber production 2 weeks after DHC treatment (*p* < 0.05; Figure 1B,C). Consequently, DHC treatment markedly alleviated fibrosis-induced skin hardness at 1 and 2 weeks post-treatment compared with normal saline treatment (*p* < 0.05; Figure 1D).

#### 2.1.2. Anti-Fibrotic Effects

**Figure 2:**
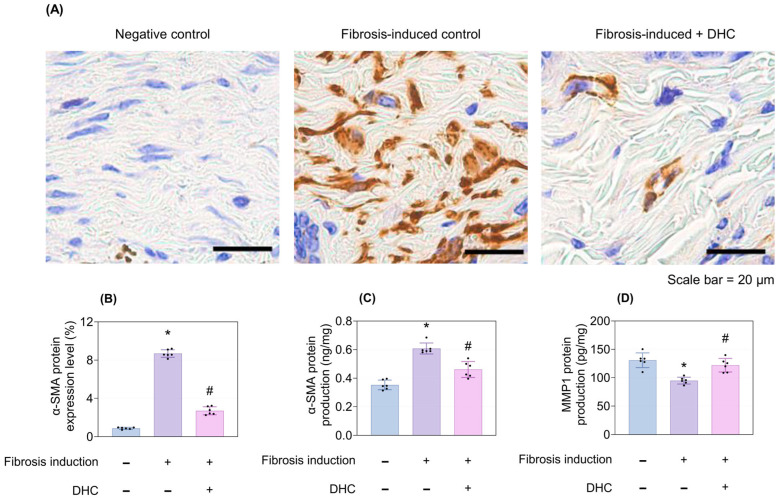
Reduction in α-SMA expression level and protein production of α-SMA and MMP1 in the in vivo mouse model. (**A**) α-SMA immunohistochemistry staining images of the negative control, fibrosis-induced control, and fibrosis-induced + DHC groups. (**B**) Quantitative analysis of α-SMA expression level, corresponding to the statistical analysis of (**A**), measured after 3 weeks of fibrosis induction followed by 2 weeks of normal saline or DHC treatment. Expression levels of (**C**) α-SMA and (**D**) MMP1 proteins, as measured by ELISA, across the three groups. Data are presented as mean ± SD; dots represent individual values. * *p* < 0.05 vs. negative control; # *p* < 0.05 vs. fibrosis-induced control. α-SMA, alpha Smooth Muscle Actin; MMP1, Matrix Metallopeptidase 1; ELISA, Enzyme-Linked Immunosorbent Assay.

Three weeks after fibrosis induction, immunohistochemistry (IHC) staining of alpha-smooth actin (α-SMA) showed a significant increase in its expression level, both visually and quantitatively (Figure 2A,B). Enzyme-Linked Immunosorbent Assay (ELISA) testing also demonstrated an upregulation of α-SMA and downregulation of metallopreoteinase 1 (MMP1) production post-fibrosis induction (Figure 2C,D). DHC treatment successfully reversed these effects, showing its anti-fibrotic potential (*p* < 0.05; Figure 2A–D).

#### 2.1.3. Anti-Inflammatory Effects on Various Cytokines

**Figure 3:**
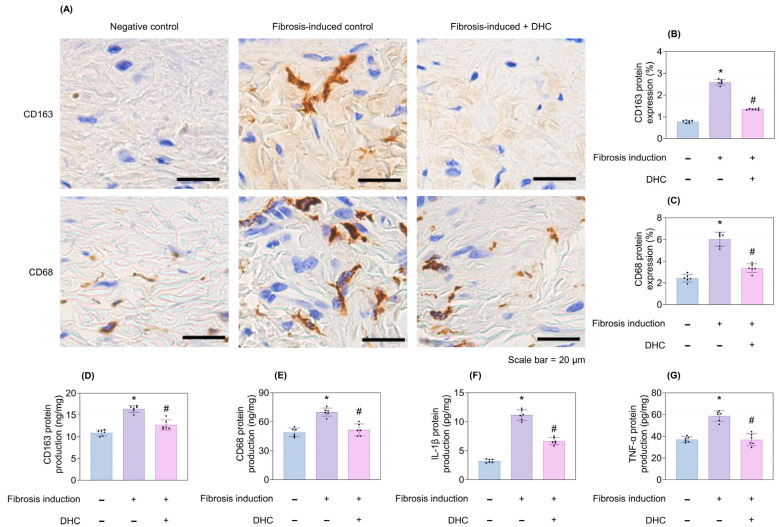
Reduction in CD163 and CD68 expression levels and protein production of CD163, CD68, IL-1β, and TNF-α in the in vivo mouse model. (**A**) CD163 and CD68 immunohistochemical staining images of the negative control, fibrosis-induced control, and fibrosis-induced + DHC groups. Quantitative analysis of (**B**) CD163 and (**C**) CD68 expression levels, corresponding to the statistical analysis of (**A**), measured after 3 weeks of fibrosis induction followed by 2 weeks of normal saline or DHC treatment. Reductions in (**D**) CD163, (**E**) CD68, (**F**) IL-1β, and (**G**) TNF-α protein levels in the DHC-treated group compared with the normal saline group, as measured by ELISA. Data are presented as mean ± SD; dots represent individual values. * *p* < 0.05 vs. negative control; # *p* < 0.05 vs. fibrosis-induced control. IL-1β, Interleukin-1β, TNF-α, Tumor Necrosis Factor alpha.

Fibrosis induction after 3 weeks promoted the expression of CD163 and CD68 via IHC staining (Figure 3A) and quantitative assessment (Figure 3B,C). It also enhanced the production of CD163, CD68, IL-1β, and TNF-α proteins on ELISA tests (Figure 3D–G). Conversely, DHC treatment successfully counteracted these effects, showing its potent anti-inflammatory properties (*p* < 0.05; Figure 3A–G).

#### 2.1.4. Antioxidant Properties

**Figure 4:**
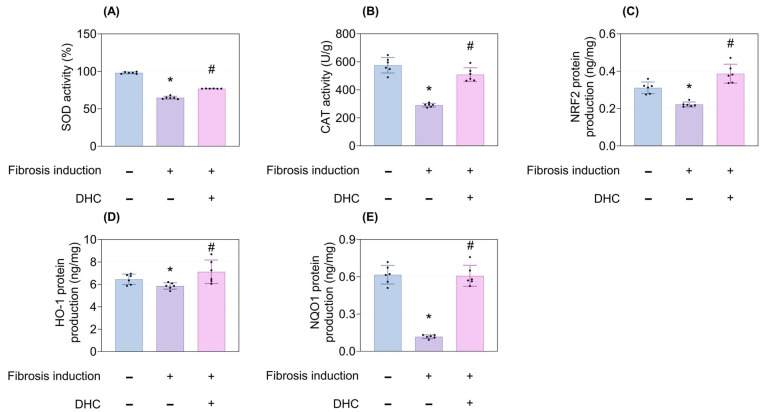
Antioxidant properties of DHC in the in vivo mouse model. (**A**) SOD activity, (**B**) CAT activity, (**C**) NRF2 production, (**D**) HO-1 production, and (**E**) NQO1 production after 3 weeks of fibrosis induction followed by 2 weeks of treatment with normal saline or DHC. Data are presented as mean ± SD; dots represent individual values. * *p* < 0.05 vs. negative control; # *p* < 0.05 vs. fibrosis-induced control. SOD, Superoxide Dismutase; CAT, Catalase; NRF2, Nuclear factor erythroid 2-related factor 2; HO-1, heme oxygenase-1; NQO1, NAD(P)H:quinone oxidoreductase 1.

Fibrosis induction markedly reduced the activity of superoxide dismutase (SOD) and catalase (CAT), as well as decreasing the production of Nuclear factor erythroid 2-related factor 2 (NRF2), Heme oxygenase-1 (HO-1), and NAD(P)H:quinone oxidoreductase 1 (NQO1) after 3 weeks. DHC treatment successfully restored the activity and production of these antioxidant enzymes, demonstrating its comprehensive redox ability (*p* < 0.05; Figure 4A–E).

### 2.2. Ex Vivo Study on UVB-Irradiated Human Skin

#### 2.2.1. Anti-Inflammatory Effects

**Figure 5:**
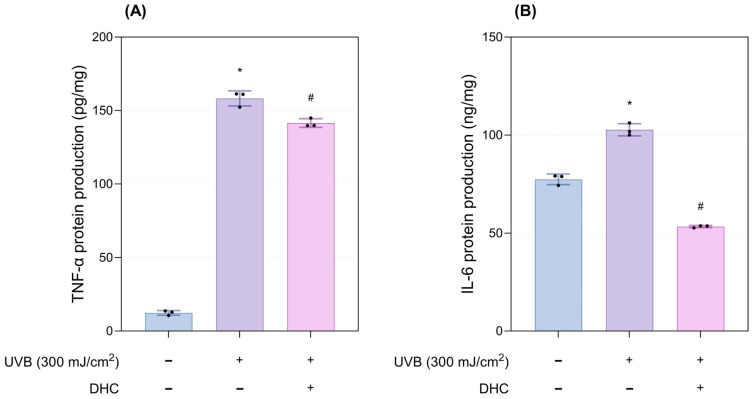
Anti-inflammatory properties of DHC in the ex vivo human skin model. (**A**) TNF-α and (**B**) IL-6 protein production 72 h after UVB exposure (300 mJ/cm^2^) and DHC treatment. Data are presented as mean ± SD; dots represent individual values. * *p* < 0.05 vs. negative control; # *p* < 0.05 vs. UVB-irradiated control. TNF-α, Tumor Necrosis Factor alpha; IL-6, Interleukin 6.

UVB irradiation significantly upregulated the production of the inflammatory cytokines TNF-α and IL-6 compared with the negative control group. In contrast, DHC treatment markedly reduced the levels of both cytokines at 72 h compared with the UVB-irradiated group (*p* < 0.05; Figure 5A,B).

#### 2.2.2. Antioxidant Properties

**Figure 6:**
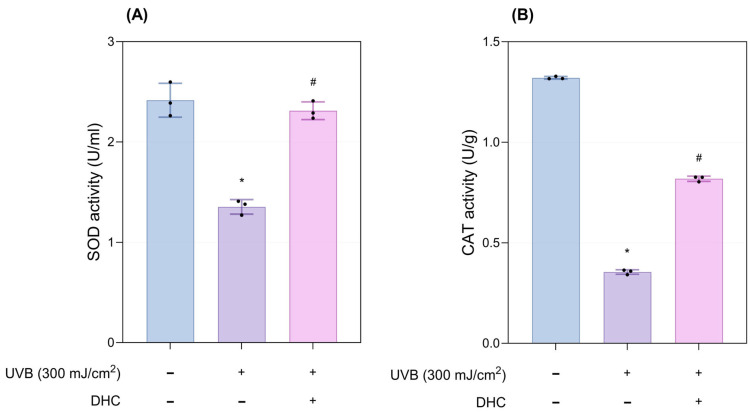
Antioxidant properties of DHC in the ex vivo human skin model. (**A**) SOD and (**B**) CAT activity 72 h after UVB exposure (300 mJ/cm^2^) and DHC treatment. Data are presented as mean ± SD; dots represent individual values. * *p* < 0.05 vs. negative control; # *p* < 0.05 vs. UVB-irradiated control. SOD, Superoxide Dismutase; CAT, Catalase.

UVB exposure markedly reduced the activities of SOD and CAT compared with the control group. Meanwhile, DHC treatment effectively preserved the activity and production of these antioxidant enzymes (*p* < 0.05; Figure 6A,B).

## 3. Discussion

Currently, HA-based formulations are widely utilized for aesthetic indications, including soft-tissue augmentation [19] and skin rejuvenation, either as standalone treatments [20] or in combination with other therapies [21]. Our previous study demonstrated that DHC exhibits broad preclinical bioactivity, characterized by biostimulatory, antioxidant, anti-inflammatory, and pigmentation-modulating effects [14]. Beyond those effects, this study demonstrates that DHC alleviated experimentally induced skin fibrosis. In this study, DHC also dampened inflammation, in which macrophage markers (CD68, CD163) and cytokines (IL-1β, IL-6, TNF-α) were markedly lower, and boosted antioxidant defenses. In summary, DHC might exert multifaceted anti-fibrotic effects by correcting the imbalances in collagen metabolism, immune signaling, and redox status caused by fibrosis.

Experimental animal models of cutaneous fibrosis are designed to reproduce sustained fibroblast activation and excessive ECM deposition [6,22]. The bleomycin-induced dermal fibrosis model, employed in this study, is one of the most widely used in vivo systems and induces dermal thickening, collagen accumulation, myofibroblast differentiation, and inflammatory cell infiltration that resemble key pathological features of fibrotic skin [23,24,25,26]. In parallel, the UVB-irradiated ex vivo human skin model in this study allows the evaluation of tissue-level responses in human skin to counteract oxidative stress commonly detected in cutaneous fibrosis [6,7,8]. Therefore, the combined in vivo and ex vivo approaches used in this study offer complementary perspectives on fibrotic remodeling. However, it’s important to note that none of the mentioned experimental skin fibrosis models can fully recapitulate the pathological background in actual skin fibrosis conditions [6,22], warranting caution when interpreting the results. In particular, the bleomycin model lacks the presence of vasculopathy usually present in sclerotic skin diseases, and is reversible with treatments that are not effective in real-life conditions [22].

Central to the pathogenesis of skin fibrosis is the activation of fibroblasts and their differentiation into myofibroblasts, resulting in increased contractility and excessive ECM deposition [6]. In this process, transforming growth factor-β (TGF-β) acts as a master pro-fibrotic cytokine. In detail, TGF-β promotes the proliferation of myofibroblast, as indicated via the upregulation of α-SMA expression, leading to enhanced collagen synthesis and higher contractility [27,28]. Additionally, TGF-β suppresses the production of ECM-degrading MMPs, one of which is MMP1 responsible for the degradation of collagen, causing the accumulation of fibrotic components [29,30,31,32]. These changes lead to dermal thickening and skin stiffening, which in turn perpetuate myofibroblast activation and survival through mechanotransduction [33]. Accordingly, our in vivo model demonstrates that DHC effectively counteracts these fibrotic processes: the reduced α-SMA expression suggests decreased myofibroblast activity, thereby lowering collagen production [34], while the upregulation of MMP1 production indicates enhanced collagen degradation [35]. Consistently, DHC-treated mice exhibited significant reductions in dermal thickness, skin hardness, and collagen content compared with untreated fibrotic controls. These effects may be attributed to the hybrid cross-linked matrix of LMW- and HMW-HA in DHC. As a viscoelastic compound, DHC provides hydration and structural support after injection [14], potentially softening the stiff ECM surrounding myofibroblasts and thereby limiting mechanotransduction-driven fibrotic activation.

Another vital aspect in skin fibrosis is chronic inflammation. Injured skin recruits immune cells (especially macrophages) that secrete IL-1β, IL-6, TNF-α, and other cytokines, reinforcing fibroblast activation and ECM deposition [1,3]. In our study, DHC markedly suppressed this inflammatory loop: CD68+ and CD163+ macrophage markers were significantly reduced in skin tissue, and production of IL-1β, IL-6, and TNF-α was curtailed. By blunting this pro-fibrotic cytokine milieu, DHC likely prevented further fibroblast recruitment and activation, contributing to its overall anti-fibrotic effect. As previously investigated in other studies, the HMW-HA presented in DHC is the key factor in regulating inflammation [14,15], suppressing the pro-inflammatory aspect of macrophages, leading to a reduction in secreted cytokines.

Oxidative stress is recognized as a driver of fibrosis: excessive ROS not only damage cells but also promote myofibroblast differentiation and collagen production [36]. Under normal conditions, NRF2 upregulates antioxidant defenses, namely SOD, CAT, HO-1, and NQO1, via the antioxidant response element to neutralize ROS [37,38]. However, persistent injury can overwhelm this system, leading to skin fibrosis [8]. In our study, we found that DHC strongly activated these components of the antioxidant system in fibrosis-induced skin. This aligns with previous laboratory studies on LMW-HA [14,16], one of DHC’s components, demonstrating its potent radical scavenging properties. LMW-HA has also been applied in cosmetic formulations to treat skin conditions related to oxidative stress, such as skin aging, wrinkles, and dryness [39]. Overall, these findings suggest that DHC can help mitigate the ROS-driven component of skin fibrosis via activation of the antioxidant system.

In comparison, most novel anti-fibrotic agents target a single pathway. For example, pirfenidone and nintedanib suppress TGF-β signaling, reducing collagen synthesis and fibroblast proliferation [40,41], while tranilast attenuates keloid and hypertrophic scarring by downregulating TGF-β1, collagen I, and α-SMA [42]. Decorin, a natural TGF-β antagonist, and losartan, an angiotensin II receptor blocker with indirect TGF-β-modulating effects, have also limited myofibroblast activation and ECM accumulation in preclinical skin fibrosis models [43,44]. In contrast, DHC may simultaneously modulate fibroblast differentiation, cytokine production, and oxidative stress. As a HA-derived product, DHC is expected to have low toxicity and high biocompatibility, positioning it as a promising therapeutic candidate for skin fibrosis pending further preclinical and clinical validation.

Several limitations should be noted. The bleomycin-induced murine model replicates key features of dermal fibrosis but does not fully reflect the complexity of human fibrotic disorders, particularly autoimmune and vasculopathic components. The exclusive use of female mice may limit the generalizability of the findings due to unassessed sex-specific responses. Only a single DHC dose and regimen were tested, precluding dose–response optimization. Although fibrosis-, inflammation-, and oxidative stress-related markers were assessed, detailed pathway analyses (e.g., TGF-β/Smad signaling or mechanotransduction pathways) were not performed. In addition, no direct comparison with established anti-fibrotic therapies was included. Finally, the ex vivo human skin model lacks systemic immune and functional vascular responses. Thus, while the findings provide supportive preclinical evidence, further mechanistic studies and well-designed clinical trials are required to confirm the translational relevance and long-term efficacy of DHC.

## 4. Materials and Methods

### 4.1. In Vivo Study

#### 4.1.1. Animal Housing and Experimental Design

Five-week-old male C57BL/6 mice (Orient Bio Inc., Seongnam-si, Republic of Korea) were acclimated for one week before experimentation [23]. To minimize experimental variability, mice of the same gender were maintained under standardized housing conditions (24 °C ± 0.5 °C; 55–65% relative humidity; 12-h light/12-h dark cycle) with free access to standard laboratory chow and water. All procedures were reviewed and approved by the Institutional Animal Care and Use Committee of Yonsei Medical Center (IACUC approval no. 2025-0152; approval date: 1 October 2025) and were conducted in full accordance with the guidelines of the Experimental Animal Center of the Yonsei University Biomedical Research Institute.

Following acclimation, mice were weighed and randomly allocated into three experimental groups: negative control, fibrosis-induced, fibrosis-induced + DHC treatment. Each group consisted of 6 mice, similar to a previous in vivo animal study on a novel filler agent [45]. To minimize variability, all experimental groups were conducted using mice within a single experiment. A bleomycin stock solution (50 mg/mL) was prepared by dissolving 25 mg of bleomycin in 500 µL of sterile normal saline, and working solutions were generated by subsequent dilution with normal saline. Dermal fibrosis was induced by daily subcutaneous injections of bleomycin sulfate (British Pharmacopoeia [BP] Reference Standard; Sigma-Aldrich, St. Louis, MO, United States; BP971) at a final concentration of 1 mg/mL (0.1 mL per injection) into the shaved dorsal skin of the animals for three consecutive weeks. All experimental groups received bleomycin except for the negative control group. All injections were performed under inhalation anesthesia with isoflurane (Hana Pharm Co., Ltd., Seoul, Republic of Korea) to minimize animal distress. After 3 weeks, 0.1 mL of the designated material was administered once via intradermal injection into the fibrotic dorsal tissue. The negative control and fibrosis-only groups received normal saline, whereas the treatment groups were administered DHC 1% (Hilowave, Higher Corporation Co., Ltd., Daegu, Republic of Korea). The animals were sacrificed two weeks after administration to evaluate the therapeutic effects.

#### 4.1.2. Dermal Thickness Measurement

Dermal thickness was evaluated in hematoxylin and eosin (H&E)-stained tissue sections. Two weeks after administration of DHC or normal saline, dorsal skin samples were fixed in 10% neutral-buffered formalin for a minimum of 24 h, processed, embedded in paraffin, and sectioned to generate paraffin-mounted slides. After deparaffinization, sections were stained with hematoxylin (S3309; Dako, Glostrup, Denmark) to visualize nuclei, rinsed under running water, and counterstained with eosin (318906; Sigma-Aldrich) to delineate cytoplasmic structures. The stained slides were subsequently washed, fully dehydrated, and coverslipped with a permanent mounting medium. Images of the epidermis and papillary dermis were acquired at 200× magnification using a light microscope (MD3000LED; Leica, Wetzlar, Germany). Dermal thickness was quantified from the captured images using ImageJ software version 1.53 (National Institutes of Health, Bethesda, MD, USA). Increased dermal thickness was interpreted as a marker of greater fibrotic severity.

#### 4.1.3. Evaluation of Collagen Fiber Production

Collagen fiber deposition was quantified in tissue sections stained with Masson’s trichrome (MT). After two weeks of administration, tissues were fixed in 10% formalin for at least 24 h, processed, embedded in paraffin, and sectioned. Deparaffinized sections were mordanted in Bouin’s solution (2010; BBC Biochemical, Mount Vernon, WA, USA) and rinsed under running water. Nuclear staining was performed with Weigert’s iron hematoxylin (hematoxylin: 4077-4425; Daejung, Siheung, Republic of Korea; ferric chloride: 660; Duksan, Ansan-si, Republic of Korea), followed by washing. Cytoplasm and muscle fibers were stained with Biebrich scarlet–acid fuchsin (Biebrich scarlet: B6008; Sigma-Aldrich; acid fuchsin: 4048-4125; Daejung), then washed again. Differentiation and decolorization were achieved using phosphomolybdic–phosphotungstic acid (phosphomolybdic acid hydrate: 84235S0410; phosphotungstic acid hydrate: 84220S0410; Junsei, Chuo-ku, Tokyo). Collagen fibers were stained with aniline blue (1087-4125; Daejung), and excess dye was removed with acetic acid. After final washing, tissues were fully dehydrated and mounted. The epidermis and papillary dermis were imaged at 200× magnification using a light microscope (MD3000LED; Leica, Wetzlar, Germany). Collagen fiber area (blue) was quantified using Zen software (ZEN 3.4 (blue edition), Carl Zeiss Microscopy GmbH, Jena, Germany), with greater area indicating higher collagen content.

#### 4.1.4. Skin Hardness Measurement

Skin hardness was assessed using a ballistometer (BLS780, Dia-Stron, Los Angeles, CA, USA), which quantifies tissue stiffness by measuring the penetration depth of a calibrated probe upon contact with the skin surface. The depth reached by the probe in the fibrotic dorsal skin was recorded for each animal. Data were expressed as the percentage increase relative to the week 0 negative control baseline, with higher values indicating greater skin hardness.

#### 4.1.5. Evaluation of Antioxidant Enzyme Activity

Collected tissues were homogenized using a TissueLyser II (Qiagen, Hilden, Germany) and centrifuged at 2000× *g* for 10 min. The supernatant, free of tissue debris, was collected for subsequent analysis of biomarker protein expression. Total protein concentrations were quantified using the Bicinchoninic Acid Protein Assay Kit (Sigma-Aldrich).

The activities of the antioxidant enzymes SOD and CAT were evaluated using the OxiTec™ SOD and CAT Assay Kits (BIOMAX, Seoul, Republic of Korea), respectively. According to the assay protocols, optical density (OD) was measured using a microplate reader (VARIOSKAN LUX, ThermoFisher Scientific, Waltham, MA, USA) to calculate enzyme activity, with lower absorbance indicating higher antioxidant activity.

#### 4.1.6. ELISA

ELISA was deployed to quantify protein levels of fibrosis-associated markers (α-SMA, MMP1), inflammation-associated markers (CD163, CD68, IL-1β, TNF-α), and antioxidant-related markers (NRF2, HO-1, NQO1) using the Mouse alpha Smooth Muscle Actin ELISA Kit (A76096, Antibodies, St. Louis, MO, USA), Mouse MMP1 ELISA Kit (EEL105, ThermoFisher Scientific), Mouse CD163 SimpleStep ELISA Kit (ab272204, Abcam, Cambridge, MA, USA), Mouse CD68 ELISA Kit (A77849, Antibodies), Mouse IL-1 beta ELISA Kit (ab197742, Abcam), Mouse TNF alpha ELISA Kit (ab208348, Abcam), Mouse Nrf2 ELISA Kit (MBS2516218, MyBioSource, San Diego, CA, USA), Mouse Heme Oxygenase 1 ELISA Kit (ab204524, Abcam), and Mouse NADH Dehydrogenase Quinone 1 (NQO1) ELISA Kit (MBS2019133, MyBioSource). All procedures were performed in accordance with the manufacturer’s protocols. Protein levels were quantified from OD measurements using standard curves on the VARIOSKAN LUX microplate reader.

#### 4.1.7. IHC Staining

Protein expression of α-SMA, CD163, and CD68 was assessed by IHC. Antigen retrieval was performed using citrate buffer (K8005, Dako, Tokyo, Japan), followed by a 2-h incubation with normal goat serum to block nonspecific binding. Primary antibodies against α-SMA, CD163, and CD68 (Anti-alpha smooth muscle Actin [1A4], ab7817; Anti-CD163 [EPR19518], ab182422; Anti-CD68 [EPR23917-164], ab283654; all from Abcam) were applied at dilutions of 1:3000, 1:200, and 1:100, respectively. Staining was performed using the Bond-X Automatic Slide Stainer (Leica Biosystems, Nussloch, Germany). Stained sections were imaged at 400× magnification using a Leica MD3000LED microscope. Protein expression of α-SMA, CD163, and CD68 was quantified as the percentage of positively stained area relative to the total tissue area using pixel-based image analysis.

### 4.2. Ex Vivo Study

#### 4.2.1. Explant Tissue Culture

The ex vivo human skin experiments were approved by the Global Medical Research Center Institutional Review Board (IRB No. GIRB-25527-MV, approval date: 13 June 2025). Residual human skin specimens were obtained from three healthy female donors of Korean descent, aged 49–55 years, who underwent facial plastic surgery. Written informed consent for the use of their tissues in research was obtained from all participants. Afterwards, the human skin tissues were trimmed to remove subcutaneous fat and rinsed with PBS to eliminate residual impurities. The excised tissues were sectioned into 1 cm × 1 cm samples and randomly allocated to three experimental groups: negative control, UVB-irradiated control, and UVB-irradiated + DHC treatment. The prepared explants were injected once with 1% DHC. Afterwards, the tissues were exposed to UVB irradiation using a UV crosslinker (BLX 312; Vilber Lourmat, Collégien, France) at a wavelength of 312 nm, delivering a cumulative dose of 300 mJ/cm^2^ once daily for three consecutive days. The emission spectrum (280–320 nm) and irradiance were continuously monitored to ensure UVB-specific exposure. Subsequently, the explants were maintained in a semi-solid culture medium at 37 °C in a humidified atmosphere containing 5% CO_2_ for up to 72 h, with culture medium replacement performed every 24 h. The semi-solid matrix was prepared by mixing 2% agar powder (Duksan) with DMEM supplemented with 10% fetal bovine serum and 1% penicillin–streptomycin (Gibco, Waltham, MA, United States) at a 1:4 ratio. Aliquots of 5 mL were dispensed into 6-well plates and allowed to solidify, providing stable support for ex vivo tissue culture. The procedures are similar to our previous study [46].

#### 4.2.2. Evaluation of Antioxidant Enzyme Activity and Pro-Inflammatory Cytokines

After 72 h of incubation, explants were processed to assess the antioxidant enzymes SOD and CAT, as described in Section 4.1.5. Pro-inflammatory cytokines IL-6 and TNF-α were quantified by ELISA using a Human IL-6 ELISA Kit (ab178013, Abcam) and a Human TNF-α ELISA Kit (ab181421, Abcam), respectively. All assays were performed in accordance with the manufacturers’ instructions.

### 4.3. Statistical Analysis

Statistical analyses were performed using IBM SPSS Statistics version 27.0. Graphs were created using GraphPad Prism version 10.2.2. Data are presented as mean ± SD. The significance level was set at *p* < 0.05. Normality was determined before each analysis. Comparisons between each group were conducted using the independent *t*-test for parametric data and the Mann–Whitney test for nonparametric data.

## 5. Conclusions

In conclusion, this study demonstrates that DHC effectively attenuates experimental cutaneous fibrosis. In a bleomycin-induced murine model, DHC reduced dermal thickening, collagen accumulation, and skin stiffness while suppressing myofibroblast activation and restoring matrix remodeling. These structural improvements were accompanied by decreased macrophage infiltration and pro-inflammatory cytokine production, as well as enhanced antioxidant enzyme activity. Consistent antioxidant effects were confirmed in UVB-irradiated human ex vivo skin. By simultaneously modulating fibroblast activation, inflammation, and oxidative stress, DHC might target multiple drivers of fibrotic remodeling. These findings support DHC as a promising multimodal therapeutic candidate for cutaneous fibrosis, warranting further clinical investigation.

## Data Availability

The original contributions presented in this study are included in the article. Further inquiries can be directed to the corresponding author.

## Abbreviations

The following abbreviations are used in this manuscript:

DHC	Dual Hyaluronic Acid Compound
HA	Hyaluronic Acid
HMW-HA	High-Molecular-Weight Hyaluronic Acid
LMW-HA	Low-Molecular-Weight Hyaluronic Acid
ECM	Extracellular Matrix
α-SMA	Alpha-Smooth Muscle Actin
MMP1	Matrix Metallopeptidase 1
IL	Interleukin
IL-1β	Interleukin-1 Beta
IL-6	Interleukin-6
TNF-α	Tumor Necrosis Factor Alpha
CD68	Cluster of Differentiation 68
CD163	Cluster of Differentiation 163
SOD	Superoxide Dismutase
CAT	Catalase
NRF2	Nuclear Factor Erythroid 2–Related Factor 2
HO-1	Heme Oxygenase-1
NQO1	NAD(P)H:Quinone Oxidoreductase 1
TGF-β	Transforming Growth Factor Beta
PDGF	Platelet-Derived Growth Factor
ROS	Reactive Oxygen Species
VEGF	Vascular Endothelial Growth Factor
IHC	Immunohistochemistry
ELISA	Enzyme-Linked Immunosorbent Assay
UVB	Ultraviolet B
PBS	Phosphate-Buffered Saline
DMEM	Dulbecco’s Modified Eagle Medium
FBS	Fetal Bovine Serum
IRB	Institutional Review Board
IACUC	Institutional Animal Care and Use Committee
SD	Standard Deviation
